# Is Environmental and Occupational Particulate Air Pollution Exposure Related to Type-2 Diabetes and Dementia? A Cross-Sectional Analysis of the UK Biobank

**DOI:** 10.3390/ijerph17249581

**Published:** 2020-12-21

**Authors:** Eirini Dimakakou, Helinor J. Johnston, George Streftaris, John W. Cherrie

**Affiliations:** 1 School of Engineering and Physical Sciences, Institute of Biological Chemistry, Biophysics and Bioengineering, Heriot-Watt University, Riccarton, Edinburgh EH14-4AS, UK; h.Johnston@hw.ac.uk (H.J.J.); john.cherrie@iom-world.org (J.W.C.); 2 Maxwell Institute for Mathematical Sciences, School of Mathematical and Computer Sciences, Heriot-Watt University, Edinburgh EH14-4AS, UK; G.Streftaris@hw.ac.uk; 3 Institute of Occupational Medicine (IOM), Riccarton, Edinburgh EH14-4AP, UK

**Keywords:** type 2 diabetes, dementia, particulate matter, air pollution, environmental exposure, occupational exposure, UK Biobank, ACE JEM, epidemiological analysis, epidemiology

## Abstract

Human exposure to particulate air pollution (e.g., PM_2.5_) can lead to adverse health effects, with compelling evidence that it can increase morbidity and mortality from respiratory and cardiovascular disease. More recently, there has also been evidence that long-term environmental exposure to particulate air pollution is associated with type-2 diabetes mellitus (T2DM) and dementia. There are many occupations that may expose workers to airborne particles and that some exposures in the workplace are very similar to environmental particulate pollution. We conducted a cross-sectional analysis of the UK Biobank cohort to verify the association between environmental particulate air pollution (PM_2.5_) exposure and T2DM and dementia, and to investigate if occupational exposure to particulates that are similar to those found in environmental air pollution could increase the odds of developing these diseases. The UK Biobank dataset comprises of over 500,000 participants from all over the UK. Environmental exposure variables were used from the UK Biobank. To estimate occupational exposure both the UK Biobank’s data and information from a job exposure matrix, specifically developed for UK Biobank (Airborne Chemical Exposure–Job Exposure Matrix (ACE JEM)), were used. The outcome measures were participants with T2DM and dementia. In appropriately adjusted models, environmental exposure to PM_2.5_ was associated with an odds ratio (OR) of 1.02 (95% CI 1.00 to 1.03) per unit exposure for developing T2DM, while PM_2.5_ was associated with an odds ratio of 1.06 (95% CI 0.96 to 1.16) per unit exposure for developing dementia. These environmental results align with existing findings in the published literature. Five occupational exposures (dust, fumes, diesel, mineral, and biological dust in the most recent job estimated with the ACE JEM) were investigated and the risks for most exposures for T2DM and for all the exposures for dementia were not significantly increased in the adjusted models. This was confirmed in a subgroup of participants where a full occupational history was available allowed an estimate of workplace exposures. However, when not adjusting for gender, some of the associations become significant, which suggests that there might be a bias between the occupational assessments for men and women. The results of the present study do not provide clear evidence of an association between occupational exposure to particulate matter and T2DM or dementia.

## 1. Introduction

Individuals are exposed to many environmental risk factors, such as air pollution, chemicals, radiation, and noise, which may impact on their health. There are several studies that indicate links between air pollution, especially ambient air particulate matter (PM), and both short-term and long-term health effects [1]. It is now widely accepted that exposure to PM may cause or exacerbate allergic respiratory diseases (e.g., asthma), pneumonia, chronic obstructive pulmonary disease (COPD) and cardiovascular disease [2], and is classified as a human carcinogen by International Agency for Research on Cancer (IARC) [3]. The cellular and molecular mechanisms underlying the toxicity of inhaled particles has been extensively investigated, and hypothesised to be driven by the stimulation of inflammation and/or oxidative stress [4].

More recently, there has also been evidence from laboratory and epidemiological studies that long-term particulate air pollution exposure is associated with diabetes and especially type-2 diabetes mellitus (T2DM) and dementia [5,6,7,8,9,10,11,12,13]. Millions of people are estimated to live with these conditions, with increasing incidence expected over coming years. Moreover, epidemiological evidence has identified that diabetes and neurodegenerative diseases involving dementia are linked and that the relationship between them may arise as a consequence of a common inflammatory mechanism [14,15]. As diabetes and dementia are linked an increased cooccurrence of those two conditions is found. T2DM may increase the risk of developing all-cause dementia, Alzheimer’s disease (AD) and vascular dementia and therefore a considerable overlap in the risk factors has been identified [16,17].

There are many occupations that are exposed to airborne particles, such as mineral dusts, metal and polymer fumes, and ultrafine particles. Some exposures in the workplace are very similar to environmental particulate pollution, mainly where there is potential exposure to combustion aerosol, such as diesel soot, or fumes. More specifically, ultrafine particles (defined as having a diameter of <100 nm) are encountered in both ambient air and at the workplace. The particles that workers may be exposed to often have similar physicochemical properties (such as size) to ambient particulate air pollution, and therefore it is a concern that exposure to such particles in an occupational setting may cause similar adverse health outcomes. Key occupations exposed to these pollutants include construction workers, tunnel workers, miners, farmers using diesel equipment, wood burners, road-patrol officers, people who work in parking lots or automobile factories, and generally workers in open areas in cities. There is evidence that occupational exposure to PM can have a negative impact on health. For example, there are studies that suggest that such exposures are related with cardiovascular diseases [18,19] or respiratory diseases [20]. In addition, from research on traffic police in a big city who were exposed to urban pollutants, there is evidence of altered levels of plasma insulin [21] and metabolism adaptation which is likely involved in the development of diabetes mellitus [22].

The UK Biobank resource offers the opportunity to investigate the association between particulate air pollution and diabetes and dementia. This is a population cohort of over 500,000 participants from all over the UK. As an individual’s job title has commonly been identified as a major determinant of workplace exposure in epidemiological studies, the development of Job–Exposure Matrices (JEMs) provides a way of characterising specific workplace exposures [23]. The ACE JEM (Airborne Chemical Exposure–Job Exposure Matrix) was originally developed to investigate workplace causes of COPD amongst participants in the UK Biobank. It covers a range of different airborne workplace pollutants, such as fumes, dust, and diesel exhaust particulates [24]. This is the largest study to date of particulate environmental and occupational exposures and T2DM and dementia.

The primary aim of this work is to use data from the UK Biobank to verify the association between environmental particulate air pollution exposure (as particulate with average aerodynamic diameter less than 2.5 μm; PM_2.5_) and T2DM and dementia, and to investigate if occupational exposure to particulates have effects similar to environmental air pollution for these diseases. It is hypothesised that as environmental exposure to particulate air pollution can cause adverse health outcomes, occupations with exposure to PM_2.5_ could cause similar adverse health effects, and in particular T2DM and dementia.

## 2. Materials and Methods 

### 2.1. Population and Study Design

Baseline data were used from the UK Biobank study (project code 42084). The UK Biobank [25] is a large population-based cohort study created to investigate how environment, lifestyle factors, and genetic background could affect an individual’s health. Approximately 9.2 million people were invited between 2006 and 2010 to attend 22 assessment centres all over UK, to obtain a sample of 502,504 adults aged between 37 and 73 years. After recruitment, participants attended an assessment centre for data collection (baseline assessment), including smoking status, alcohol consumption, body mass index (BMI), and physical activity, after providing a written informed consent and then they were followed-up. UK Biobank provides generally detailed information about lifestyle, physical measures, genetics, and imaging and is globally accessible to approved researchers who are undertaking health-related research that is in the public interest .

### 2.2. Disease Categories

**Figure 1:**
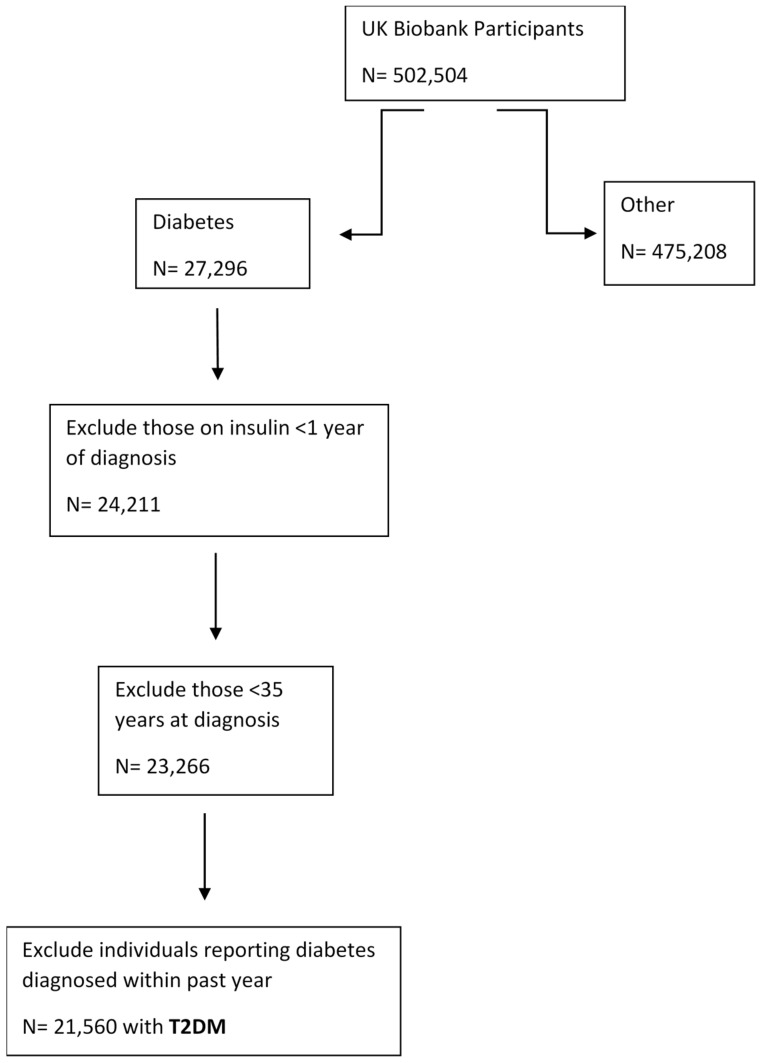
Flow chart demonstrating how the population sample was identified for type-2 diabetes mellitus (T2DM).

Participants provided a self-report disease status during a touchscreen questionnaire. Diabetes data were retrieved from field 2443 with the description “Diabetes diagnosed by doctor”. In order to reduce the likelihood of type-1 diabetes mellitus (T1DM) and other forms of diabetes, we excluded those on insulin for less than a year from diagnosis, anyone < 35 years old at diagnosis of diabetes and individuals reporting diabetes diagnosed within past year [26]. After the exclusion we obtained the participants with presumptive T2DM (*n* = 21,560) (flow chart in Figure 1).

Dementia patients were identified using the field 41202 (Diagnoses-main ICD10; International Classification of Diseases 10th Revision), which is a summary of the main/primary diagnosis codes a participant has had recorded across all their hospital inpatient records. Only codes corresponding to dementia were selected (Alzheimer’s disease, vascular dementia, frontotemporal dementia, dementia in other diseases, and unspecified dementia) to obtain the participants diagnosed with dementia (*n* = 534).

### 2.3. Environmental and Occupational Variables

Environmental exposure variables were used from the UK Biobank. More specifically, information on exposure to PM air pollution was modelled for the year 2010 using a Land Use Regression (LUR) model as part of ESCAPE (European Study of Cohorts for Air Pollution Effects) project [27]. PM_2.5_ is comprised of smaller particles than PM_10_ which are generally considered more relevant to adverse health effects, and so this is the focus of this study.

The job code mentioned at the time of the baseline assessment was used. The participants were asked about their job in the verbal interview, depending on whether they first stated they were currently employed or self-employed in the touchscreen interview. In addition, the job codes for a subset of the participants for their full working career were provided during an online follow-up along with other variables (year job started, year job ended), and these data were used to reconstruct lifetime exposure estimates. This subgroup of participants responded to the Online Work Environment Questionnaire of the UK Biobank, which was sent out to all the UK Biobank participants. Finally, three self-reported exposure variables from the UK Biobank dataset were used (workplace very dusty, workplace full of chemical or other fumes and workplace had a lot of diesel exhaust) for further examination of the occupational exposure.

### 2.4. Occupational Variables and ACE JEM

We extracted information from the ACE JEM on workplace exposure to dust, fumes, diesel exhaust, mineral dust, and biological dust, which uses the UK Standard Occupational Classification (SOC) 2000 system and combined this with the information from UK Biobank about each individual’s occupation. The ACE JEM provides the proportion (P) of workers that were estimated to be exposed to each of the pollutants (< 5%, 5–19%, 20–49%, and ≥ 50%), within a given SOC code, and the level or intensity of exposure (L) as a typical average per day or weekly exposure (categorised as “not exposed”, “low”, “medium”, or “high”). As we did not want to weight the categories differently, and there was a need to reflect the true exposures, a new scale was created for the intensity of exposure, by using a factor of 5, and not the original scale mentioned at the ACE JEM (Table S1).

To obtain a combined measure of exposure including both measures, we used the product of P and L (P × L), which was computed by multiplication of the values in each category of P and L. Therefore, each participant corresponds to a (P × L) score according to their occupational exposure at each of the five agents (dust, fumes, diesel, biological dust, and mineral dust). 

### 2.5. Covariates

Following a review of known risk factors for T2DM and dementia, we selected appropriate variables from the UK Biobank for inclusion in the analysis. Baseline measurements of socioeconomic and demographic covariates (age as years, sex, ethnic background, BMI, Townsend Deprivation Index of residential area, diet, physical activity, family history of the disease, or other conditions) were included as covariates in the model. Where there were measurements made on several occasions (baseline assessment, repeat assessments), means were calculated when appropriate. Physical activity was considered and included in the model, by selecting a variable which calculated the total physical activity MET (Total Metabolic Equivalent Task) in minutes per week, for all activity including walking, moderate and vigorous activity. Smoking (tobacco smoking) and alcohol consumption (frequency of drinking alcohol) data items were not included in the analysis, due to the high percentage of missing values in the dataset.

### 2.6. Statistical Analyses

Descriptive analysis was undertaken on the prevalence of the diseases and how this was distributed between the different groups of the covariates. To investigate the association between environmental pollution and T2DM and dementia, univariable and multivariable logistic regression was performed. The first model introduced the unadjusted association between environmental exposure and the outcome of interest (the two diseases). The second model was adjusted for several factors. Univariable and multivariable logistic regression were also performed, in order to explore if occupational exposure is associated to the two diseases. The models adjusted for all included variables for each disease (PM included), using DAGs (Directed Acyclic Graphs) developed for the diseases of interest (Figures S1 and S2) and then stepwise regression. The occupational variable (P × L) was recategorised into two levels, with level 1 representing no exposure and level 2 representing some exposure. This recategorisation was chosen because the ACE JEM assignments are probably reliable for highly exposed jobs and for jobs assigned as unexposed, while the assignments for medium exposed and low exposed are less reliable [23]. This is a complete case analysis, as participants with missing data were excluded (Table S2) and only participants with full data records were included in the analysis. All analyses were conducted using the statistical software R version 3.6.2 [28].

### 2.7. Sensitivity Analyses

Different approaches were considered for sensitivity analysis. The associations between T2DM and dementia and occupational exposure were explored further in four different ways. Approach 1: We performed the same analysis, but without adjusting for gender. Approach 2: To explore further the occupational association, and as individuals are exposed to a combination of pollutants [29], we recategorised the exposure (P × L) to every agent in three levels (high, medium, and low) according to their (P × L) score percentages, and then kept the maximum occupational exposure across all five agents (dust, fumes, diesel, mineral, and biological dust). Then, we recategorised further in two new levels (high and medium exposure together, low exposure separately) and created a new occupational variable ((PL-max), which was then further considered to investigate if this recategorisation altered the outcome. Approach 3: Self-reported workplace exposures were selected from the UK Biobank (workplace very dusty, workplace with fumes, or workplace with diesel engine exhaust) and entered in the model instead of the (P × L) variable, to investigate further the relationship between occupational exposure, as reported from the participants, and the two diseases. Approach 4: A subgroup of 120,299 participants for whom there was a full occupational history was investigated, after calculating the cumulative exposure through their occupational lifetime, based on the length of time for which they carried out a specific job, to check the consistency of our results.

## 3. Results

### 3.1. Characteristics of the Study Population

Of the 502,504 UK Biobank participants, after excluding those who were likely not to have T2DM, there were 21,560 participants with T2DM and 534 cases of dementia (Table 1). From the total number of the participants, 273,382 (45.6%) were male and 229,122 (54.4%) were female. From the participants diagnosed with dementia, 309 were males and 225 females and from the participants with T2DM, 13,036 were male (60.5% of the T2DM population), and 8524 were female (39.5% of the T2DM population). The age ranged from 37 to 73, with the modal category being 57–66 years old at the recruitment, with 43.9% self-reporting as white ethnic background (94.2%). Around 20% of the UK Biobank population was taking medication for blood pressure and cholesterol. Twelve percent had changed their diet because of illness and 30% for other reasons. As expected, because of the nature of both diseases investigated in this study, the proportion of diseased people increased at ages above 56 years. According to the Townsend Deprivation Index, which is used as a proxy for SES (socioeconomic status), the prevalence of both diseases increased as the deprivation increased. Most of the cases for both diseases had a BMI over 25, which corresponds to them being overweight or obese. Fewer cases were observed, as the reported physical activity levels increased. 

### 3.2. Association between Particulate Air Pollution (PM_2.5_) and T2DM and Dementia

The results of analyses to investigate the association between environmental particulate exposure (PM_2.5_) and T2DM and dementia are shown in Table 2. Univariable analysis suggested that PM is strongly positively related to both diseases (with an Odds Ratio (OR) of 1.13 (1.12–1.15) per 1 μg/m^3^ increase in annual average PM_2.5_ for T2DM and 1.18 (1.09–1.28) per unit for dementia). The results from the multivariable logistic regression are also displayed in Table 2, showing a summary of ORs for T2DM and dementia when other factors are also included in the model. In order to select a parsimonious model that fitted the data well, the covariates for the multivariable analysis were selected by creating directed acyclic graphs (DAGs, shown in Figures S1 and S2) and then following a stepwise regression model. Based on the DAGs we excluded the blood pressure (BP) variable, as it was judged to be on the causal pathway of PM_2.5_ and both diseases. This was followed by stepwise regression for determining the best model with the smallest AIC [30]. For T2DM this model included age, sex, ethnicity, Townsend deprivation index, BMI, dietary changes, physical activity, and parental history of diabetes, and for dementia age, sex, Townsend deprivation index, dietary changes, physical activity, and parental history of dementia. In the adjusted models, PM_2.5_ was associated with an OR of 1.02 (95% CI 1.00 to 1.03) per unit PM_2.5_ for developing T2DM, while the corresponding OR for dementia was 1.06 (95% CI 0.96 to 1.16). In both adjusted models the odds of having the disease increased as the deprivation index increased (Table 2) and decreased for higher levels of physical activity. 

### 3.3. Association between Occupational Exposure and T2DM and Dementia

The results of analyses to investigate the association between occupational exposure and T2DM and dementia are displayed in Table 3 and Tables S3 and S4 in the Supplementary Material. There were five different occupational exposures (dust, fumes, diesel exhaust, mineral, and biological dust) and the results of the separate logistic regression models for each pollutant are displayed in this table. In the unadjusted (univariable) models, associations were evident between four out of the five agents (dust, fumes, diesel, and mineral dust) and T2DM. Note that the range of the diesel exhaust occupational exposure (P × L) values are between 0.000 and 0.225, which implies that the interpretation of the OR in Table 3, as per unit increase may not be reliable. However, in the adjusted models where other factors are also included, the OR confidence intervals shows non-significant association between most exposures and T2DM, and there were no detectable associations between any of the exposures and dementia. When the interaction between occupational exposure and sex was included in the adjusted model, the analysis showed significant differences in the impact of some of the occupational exposures on T2DM, with a negative impact for males in some cases (Table S5). For that reason, a sensitivity analysis was conducted, to investigate differences in the associations without including gender in the model.

### 3.4. Sensitivity Analyses

The logistic regression results for the association between occupational exposure and the two diseases are shown in Table 4, without adjusting for gender. There was a positive association between fumes and both diseases (with OR (95% CI): 1.24 (1.17–1.31) per 1 μg/m^3^ increase in PM_2.5_ for T2DM and 1.57 (1.06–2.27) per 1 μg/m^3^ increase in PM_2.5_ for dementia and a positive association between diesel exhaust and mineral dust and T2DM (OR (95% CI): 1.31 (1.22–1.41) and 1.11 (1.04–1.17) accordingly).

A newly created occupational variable (PL-max), which represents the maximum occupational exposure across all five substances, suggested that, when we did not adjust for sex, there was a significant association between occupational PM exposure and T2DM (1.10 (1.05–1.15)) and dementia (1.40 (1.00–1.95)). However, when we also adjusted for sex, the association for both diseases became non-significant. This suggests a dependence between the occupational exposure variable (P × L) and gender, and that (P × L) may be a proxy for sex. For all of the self-reported occupational exposures, logistic regression indicated that there were no significant associations with disease status (Table 5).

From the subgroup analysis of the occupational history of the 120,299 participants (Table 6), the point estimates and confidence intervals seem to be generally consistent with the results from the whole dataset. The multivariable analysis with this subgroup of the participants suggested that although we have a lifetime estimate of occupational exposure, which should provide a more accurate picture of exposure, the ORs were not statistically different from unity.

## 4. Discussion

Epidemiological evidence linking long-term, environmental particulate air pollution to adverse health effects, such as T2DM and dementia, has gradually increased over recent years [31,32,33]. Our study is consistent with findings from other research, with a significant association between PM_2.5_ and T2DM (OR (95% CI): 1.02 (1.00–1.03)) per unit exposure [34,35]. A systematic review and meta-analysis by Yang et al., showed significant associations of PM_2.5_ and T2DM prevalence (1.08 (1.04–1.12) per 10 μg/m^3^ increment [35]. Our study is also consistent with other studies that link PM_2.5_ and dementia [36,37,38] as the size of the effect is similar, although because of the relatively small number of people with dementia in the UK Biobank (534 cases) it probably does not have sufficient power to detect an association of this magnitude. Other studies have adjusted for other covariates which makes comparing effect sizes difficult. According to Lee et al., the adjusted hazard ratio (HR) of hospitalization with the disease was 1.05 (1.04–1.05) per 1 μg/m^3^ increase in annual PM_2.5_ [37] and according to Chen et al. a 1.04 (1.03–1.05) HR was observed, for every interquartile-range increase in PM_2.5_ exposure [38]. Moreover, a recent report on dementia, by Livingston et al. clearly states that air pollution is a risk factor for dementia [39]. Interestingly, there are other studies that suggest that air pollution is positively associated with cognitive decline with bigger effects, as an interquartile range increase in PM_2.5_ gave an OR of 1.16 (1.05–1.27), but these studies seem to be of smaller population size, and investigated mild cognitive impairment (MCI), which is an early stage of disease and not dementia *per se* [40]. A study of the UK Biobank, by Cullen et al. showed a weak association between air pollution and cognitive performance [41]. A meta-analysis by Fu et al. indicated that long-term PM_2.5_ exposure was associated with an increased risk of dementia (1.16 (1.07–1.26) per 10 μg/m^3^ PM_2.5_), although their meta-analysis for AD (Alzheimer’s disease) was not significant (3.26 (0.84–12.74)) [33]. All the other factors in the environmental models, for both diabetes and dementia, produced results consistent with what is already known in the literature. For example, for the most deprived areas the risk for both diseases was increased [42,43], and the risk was higher for T2DM for certain ethnicities [44,45] and physical activity had a protective effect for T2DM [46] (Table 2). However, these ORs need to be interpreted with caution, because of the issues described by Westreich et al. [47], as effect measures for secondary risk factors can be biased if they are interpreted in the same way as the primary risk factor. 

To the best of our knowledge, although there are many studies that examine the relationship between occupational exposures such as night-shift work, job strain, or specific substances such as solvents and diabetes [48,49,50] or dementia [51,52,53,54,55], this is the first study to investigate an association between occupational exposure to PM that are similar to environmental air pollution exposure and both health outcomes. We relied on the ACE JEM to assign exposure probability and intensity to jobs, although from our earlier work it is likely that these assignments were most reliable for highly exposed jobs and for jobs assigned as unexposed [23]. We chose to recategorise into exposed and unexposed to attempt to minimise the potential for misclassification in the analysis. In the multivariate analyses the risks for occupational exposure to dust and mineral dust produced significant ORs for T2DM less than one, which do not seem biologically plausible. All the other comparisons yielded ORs that were not significantly different from one. Our findings for diesel exhaust are consistent with previous work by Koeman et al., which found among other exposures, that there was no association between motor exhaust and dementia, although Meo et al. found an association between motor exhaust and T2DM [56,57]. Moreover, when occupational exposure and environmental exposure were both in our model, we observed PM_2.5_ changes that suggest that these two exposures may not be completely independent from each other (Tables S3 and S4).

There are a number of limitations in the current study that could mask associations between occupational PM exposure and effects on the brain or diabetes mellitus [58]. The analysis was cross-sectional; therefore, causality cannot be determined. It is as viewing a snapshot of the population at a certain point in time, thus the identification of the participants is based on the absence or presence of the exposure and the presence of the exposure of the disease. Another weakness of this study was the lack of historical information surrounding exposure (both environmental and occupational). Moreover, the occupational analysis was based on a JEM, and the simplicity of this tool cannot adequately account for the variability in exposure between workers or between workplaces. JEMs are also generally not good at identifying small effects, especially JEMs like the ACE JEM that are generated from subjective information rather than objective measurements [59]. From our earlier evaluation of the ACE JEM it was observed that the assignments for medium and low exposed jobs were less reliable than for high and unexposed jobs [23]. Moreover, there may be problems in the JEM’s coding for men and women, as it seems there is inconsistency between the assessments for the two gender. A further potential limitation of the study was the definition of diabetes in UK Biobank, as this variable was self-reported as just diabetes, which necessitated inference to identify presumptive T2DM participants. Another limitation of this study was that information about smoking and alcohol could not been used because of a high percentage of missing values. We used the Townsend deprivation index as a proxy for these and other lifestyle factors that may influence the risk of the diseases studied, but this measure suffers from lack of specificity.

Our study has some major strengths, the UK Biobank provides a large data source that is geographically diverse, and allows adjustment for multiple environmental, social, and occupational related factors. Moreover, this study is based on both the self-reported information in UK Biobank’s and ACE JEM’s information to create a more objective occupational exposure measure on which to draw conclusions. 

Another strength of this study is the multiple approaches used in the sensitivity analysis (Table 4, Table 5 and Table 6) to investigate the relationship between occupational exposure and the health outcomes. Firstly, the same analysis was performed without adjusting for gender (Table 4), as sex seemed to be a proxy for occupational exposure and that the impact of (P × L) may reflect the impact of gender. From the logistic regression without adjusting for sex the results suggested that fumes had a significant association with both diseases and also diesel and mineral dust occupational exposure may have an impact on the risk of T2DM. Those significant results are consistent with studies that link ultrafine particles with the diseases [60] and occupations such as traffic policemen that have to deal with diesel exhaust and the T2DM outcome [22]. A possible explanation for the apparent difference in risk between male and female when the sex interaction is included in the model, could be that the ACE JEM provides unreliable information for jobs typically carried out by men and women. There may be a bias in the way that typical male jobs were assessed compared to typical female jobs. Secondly, to examine the maximum exposure of all the substances to which a participant could be exposed, we created the variable PL-max (Table 5). After running the logistic regression models, while adjusting for sex, we obtain non-significant results. Although, if we hypothesize that (P × L) exposure assignments and sex were interrelated and exclude sex from the model there was a significant association between occupational exposure to PM and both T2DM and dementia (OR (95% CI): 1.10 (1.05–1.15) for T2DM and (OR (95% CI): 1.40 (1.00–1.95) for dementia). So, although the results from the main analysis cannot detect an association between occupational exposure and the diseases, by investigating an extreme workplace exposure measure, a significant association was observed. To examine further the three main occupational exposures (dust, fumes, and diesel exhaust), we also considered the self-reported workplace exposure variables in the UK Biobank dataset. These analyses did not suggest any relationship between self-reported exposure and the diseases (Table 5), which is consistent with the main results of this study. Lastly, the sensitivity analysis performed in a subgroup of UK Biobank participants with a full working history, provides a more reliable estimate of lifetime occupational PM exposure. The association between occupational exposure and T2DM and dementia remained undetectable in this population (Table 6). 

## 5. Conclusions

There is no strong evidence from the present study for an association between occupational exposure to particulates that are similar to environmental air pollution and T2DM or dementia, although our analysis suggests an association between particulate air pollution exposure and T2DM. Additionally, we did not find a significant association between PM_2.5_ and dementia, and this could be because of the relatively low dementia prevalence in the cohort. Our findings were verified in the subgroup of participants for whom we had more complete estimates of cumulative working life exposures. However, when the analysis was carried out without adjusting for sex, some of the associations become significant, and this suggests there may be differences between the JEM assignments for jobs typically carried out by men and women. 

If there is an association between occupational exposure to PM and T2DM or dementia, it is likely to be small, which is consistent with the observations for PM air pollution. Given that people are exposed to air pollution continuously over the whole lifetime while occupational exposure is for only a fraction of that time, the magnitude of the impact related to occupational exposure is not expected to be larger than the environmental impact. More reliable occupational information for a larger number of participants in the UK Biobank, in combination with an improved JEM or an alternative way of estimating workplace PM exposures, would help in further investigating the association between occupational PM exposure and T2DM and dementia.

## Figures and Tables

**Table 1:** Descriptive statistics.

Baseline Variable	Grouping	All Subjects			Dementia			Type 2 Diabetes Mellitus		
		*n*	% (with NA’s)	%	*n*	% (Out of 502,504)	% (Out of the Diseased)	*n*	% (Out of 502,504)	% (Out of the Diseased)
**Sex**										
	All	502,504	100	100	534	0.11	100	21,560	4.30	100
	Male	273,382	45.60	45.60	309	0.06	57.87	13,036	2.60	60.46
	Female	229,122	54.40	54.40	225	0.04	42.13	8524	1.70	39.54
	Missing	-	-	-	-	-	-	-	-	-
**Age recruitment**										
	All	502,504	100	100	534	0.11	100	21,560	4.30	100
	(36–46)	77,177	15.36	15.36	30	0.01	5.62	1132	0.23	5.25
	(46–56)	151,241	30.10	30.10	81	0.02	15.17	4704	0.94	21.82
	(56–66)	220,407	43.86	43.86	275	0.05	51.50	11,597	2.31	53.79
	(66–73)	53,679	10.68	10.68	148	0.03	27.71	4127	0.82	19.14
	Missing	-	-	-	-	-	-	-	-	-
**Ethnicity**										
	(All-Missing)	501,606								
	All	502,504	100	100	533	0.11	100	21,559	4.13	100
	White	472,695	94.24	94.24	504	0.10	94.55	18,828	3.75	87.33
	Mixed	2958	0.59	0.59	4	0.00	0.75	137	0.03	0.64
	Asian	11,456	2.28	2.28	5	0.00	0.94	1442	0.28	6.69
	Black	8061	1.61	1.61	10	0.00	1.87	684	0.13	3.17
	Others	6436	1.28	1.28	10	0.00	1.87	468	0.09	2.17
	Missing	898	0.17	-	1	0.00	-	1	0.00	-
**SES**										
	(All-Missing)	501,881								
	All	502,504	100	100	533	0.09	100	21,529	4.28	100
	1	100,658	20.03	20.10	85	0.02	15.95	3215	0.64	14.93
	2	100,098	19.92	19.90	71	0.01	13.32	3586	0.71	16.66
	3	100,382	19.98	20.00	96	0.02	18.01	3978	0.79	18.48
	4	100,367	19.97	20.00	116	0.02	21.76	4499	0.89	20.89
	5	100,376	19.97	20.00	165	0.03	30.96	6251	1.24	29.04
	Missing	623	0.12	-	1	0.00	-	31	0.01	-
**BMI**										
	(All-Missing)	499,503								
	All	502,504	100	100	517	0.10	100	21,345	4.24	100
	< 18.5	2374	0.47	0.50	3	0.00	0.58	19	0.00	0.09
	18.5–24.9	157,631	31.37	31.60	144	0.03	27.85	2049	0.41	9.60
	25–29.9	214,485	42.68	42.90	230	0.05	44.49	7276	1.45	34.09
	≥ 30	125,013	24.88	25.00	140	0.03	27.08	12,001	2.40	56.22
	Missing	3001	0.60	-	17	0.00	-	215	0.04	-
**BP**										
	(All-Missing)	498,639								
	All	502,504	100	100	524	0.10	100	21,430	4.26	100
	No	387,531	77.12	77.7	325	0.06	62.02	7308	1.45	34.10
	Unknown	637	0.13	0.10	3	0.00	0.57	20	0.00	0.09
	Yes	110,525	21.99	22.20	196	0.04	37.40	14,102	2.81	65.80
	Missing	3811	0.76	-	10	0.00	-	130	0.00	-
**Diet changes**										
	(All-Missing)	501,717								
	All	502,504	100	100	533	0.11	100	21,560	4.30	100
	No	296,798	59.06	59.20	272	0.05	51.03	5474	1.09	25.39
	Unknown	1443	0.29	0.30	4	0.00	0.75	78	1.02	0.36
	Yes, illness	57,550	11.45	11.50	132	0.03	24.77	12,164	2.43	56.42
	Yes, other	145,826	29.02	29.10	125	0.02	23.45	3844	0.77	17.83
	Missing	887	0.18	-	1	0.00	-	-	-	-
**Physical activity**										
	(All-Missing)	490,724								
	All	502,504	100	100	507	0.10	100	20,610	4.10	100
	Low	163,988	32.63	33.40	232	0.05	45.76	8636	1.76	41.90
	Moderate	203,130	40.42	41.40	161	0.03	31.75	7890	1.61	38.28
	High	123,606	24.60	25.20	114	0.02	22.49	4084	0.83	19.82
	Missing	11,780	2.34	-	27	0.00	-	950	0.19	-

NA: not applicable (missing values), SES: sosioeconimic status, BMI: body mass index, BP: blood pressure.

**Table 2:** Associations between T2DM and dementia and particulate air pollution (PM_2.5_) exposure and other potential risk factors.

	T2DM		Dementia	
	OR (95% CI) **	OR (95% CI) **	OR (95% CI) **	OR (95% CI) **
	Univariable Model 1	Multivariable * Model 2	Univariable Model 1	Multivariable * Model 2
**PM_2.5_**	**1.13 (1.12–1.15)**	**1.02 (1.00–1.03)**	**1.18 (1.09–1.28)**	1.06 (0.96–1.16)
**Sex** (female) ^a^				
Male	**1.88 (1.82–1.93)**	**1.83 (1.77–1.89)**	**1.64 (1.38–1.95)**	**1.55 (1.28–1.89)**
**Age**	**1.06 (1.06–0.07)**	**1.07 (1.07–1.08)**	**1.09 (1.08–1.11)**	**1.08 (1.07–1.10)**
**Ethnic background** (White)				
Asian	**3.47 (3.28–3.68)**	**3.80 (3.52–4.09)**	-	-
Black	**2.24 (2.06–2.42)**	**1.64 (1.48–1.81)**	-	-
Mixed	1.17 (0.98–1.38)	**1.38 (1.11–1.70)**	-	-
Other	**1.89 (1.72–2.08)**	**1.64 (1.48–1.81)**	-	-
**Townsend deprivation** (1)				
2	**1.13 (1.07–1.18)**	**1.06 (1.01–1.12)**	0.84 (0.61–1.15)	0.76 (0.54–1.08)
3	**1.25 (1.19–1.31)**	**1.13 (1.07–1.19)**	1.13 (0.85–1.52)	1.02 (0.74–1.41)
4	**1.42 (1.36–1.49)**	**1.18 (1.11–1.24)**	**1.37 (1.04–1.82)**	**1.38 (1.02–1.89)**
5 (most deprived)	**2.02 (1.94–2.11)**	**1.37 (1.29–1.44)**	**1.95 (1.50–2.54)**	**1.79 (1.31–2.46)**
**BMI** (< 18.5)				
≥ 30	**13.15 (8.64–21.43)**	**12.53 (7.64–22.44)**	-	-
25–29.9	**4.35 (2.85–7.08)**	**4.70 (2.86–8.42)**	-	-
18.5–24.9	**1.63 (1.07–2.66)**	**2.23 (1.35–3.99)**	-	-
**Dietary changes** (No)				
Unknown	**3.05 (2.40–3.80)**	**2.08 (1.52–2.77)**	3.03 (0.93–7.12)	1.76 (0.29–5.67)
Yes, because of illness	**14.26 (13.80–14.75)**	**11.22 (10.81–11.65)**	**2.51 (2.03–3.08)**	**1.86 (1.46–2.35)**
Yes, because of other	**1.44 (1.38–1.50)**	**1.36 (1.30–1.43)**	0.94 (0.75–1.15)	0.96 (0.75–1.20)
**Physical activity** (Low)				
High	**0.61 (0.59–0.64**	**0.76 (0.73–0.79)**	**0.94 (0.75–1.15)**	**0.96 (0.75–1.20)**
Moderate	**0.73 (0.70–0.75)**	**0.88 (0.85–0.91)**	**0.94 (0.75–1.15)**	**0.96 (0.75–1.20)**
**Father’s history** (No)				
Do not know	**1.71 (1.62–1.80)**	**1.23 (1.16–1.31)**	**2.23 (1.68–2.90)**	**1.65 (1.20–2.23)**
Prefer not to answer	**1.86 (1.34–2.52)**	1.07 (0.66–1.65)	3.28 (0.54–10.20)	1.98 (0.29–7.65)
Yes	**2.35 (2.27–2.44)**	**2.37 (2.27–2.48)**	1.23 (0.82–1.77)	1.30 (0.85–1.91)
**Mother’s history** (No)				
Do not know	**1.63 (1.53–1.74)**	1.06 (0.97–1.15)	**1.67 (1.10–2.43)**	0.99 (0.60–1.53)
Prefer not to answer	**1.78 (1.24–2.47)**	0.93 (0.54–1.53)	**6.00 (1.49–15.72)**	**5.07 (1.10–15.59)**
Yes	**1.06 (1.03–1.09)**	**1.08 (1.05–1.12)**	1.03 (0.77–1.37)	1.07 (0.77–1.45)

* Adjusted regression OR of multivariable logistic regression analysis. ^a^ Reference category is shown in brackets. ** Significant associations in bold.

**Table 3:** Associations between T2DM and dementia and occupational exposure.

T2DM		
(P × L) ***	**OR (95% CI) Univariable**	OR (95% CI) Multivariable *
Dust ^a^	**1.11 (1.00–1.22)**	**0.93 (0.88–0.98)**
Fumes ^a^	**1.60 (1.29–1.96)**	1.01 (0.95–1.07)
Diesel ^a^	**27.69 (13.47–55.35)**	1.07 (0.99–1.15)
Mineral Dust ^a^	**1.35 (1.16–1.57)**	**0.91 (0.86–0.97)**
Biological Dust ^a^	0.78 (0.78–1.13)	0.95 (0.89–1.02)
**DEMENTIA**		
(P × L) ***	**OR (95% CI) Univariable**	OR (95% CI) Multivariable **
Dust ^a^	1.51 (0.73–2.72)	1.02 (0.71–1.45)
Fumes ^a^	1.80 (0.26–5.98)	1.25 (0.84–1.84)
Diesel ^a^	1.22 (0.00–414.36)	0.98 (0.56–1.62)
Mineral Dust ^a^	1.69 (0.49–4.08)	1.02 (0.66–1.51)
Biological Dust ^a^	**2.74 (1.07–5.57)**	0.97 (0.58–1.52)

* Model is adjusted for PM_2.5_, age, sex, ethnicity, BMI, Townsend deprivation score, dietary changes, physical activity, and parental history of diabetes. ** Model is adjusted for PM_2.5_, age, sex, Townsend deprivation score, dietary changes, physical activity, and parental history of dementia. *** Significant associations in bold. ^a^ Level (1) Reference level: no exposure—Level (2): Exposure; estimates given for level (2).

**Table 4:** Sensitivity analysis for occupational exposure and T2DM and dementia, without adjusting for sex.

	T2DM	Dementia
(P × L) ***	OR (95% CI) Multivariable *	OR (95% CI) Multivariable **
**Dust ^a^**	1.01 (0.96–1.06)	1.14 (0.79–1.61)
**Fumes**	**1.24 (1.17–1.31)**	**1.57 (1.06–2.27)**
**Diesel**	**1.31 (1.22–1.41)**	1.26 (0.72–2.05)
**Mineral Dust**	**1.11 (1.04–1.17)**	1.26 (0.83–1.86)
**Biological Dust**	**0.90 (0.84–0.96)**	0.95 (0.55–1.44)

* Model is adjusted for PM_2.5_, age, ethnicity, BMI, Townsend deprivation score, dietary changes, physical activity, and parental history of diabetes. ** Model is adjusted for PM_2.5_, age, Townsend deprivation score, dietary changes, physical activity, and parental history of dementia. *** Significant associations in bold. ^a^ Level (1) Reference level: no exposure—Level (2): Exposure; estimates given for level (2).

**Table 5:** Sensitivity analysis for occupational exposure (PL-max) and self-reported exposures and T2DM and dementia.

	T2DM *	Dementia **
	OR (95% CI)	OR (95% CI)
(PL-max) ***	**1.10 (1.05–1.15)**	**1.40 (1.00–1.95)**
**(PL-max)**	0.96 (0.91–1.01)	1.20 (0.85–1.69)
**Workplace very dusty**	1.01 (0.93–1.09)	1.04 (0.48–2.24)
**Workplace with fumes**	0.95 (0.87–1.03)	1.15 (0.48–2.54)
**Workplace with diesel**	0.99 (0.90–1.09)	1.54 (0.58–3.67)

* Adjusted for age, sex, PM_2.5_, ethnicity, Townsend deprivation index, BMI, dietary changes, physical activity, paternal history of diabetes. ** Adjusted for age, sex, PM_2.5_, Townsend deprivation index, dietary changes, physical activity, paternal history of dementia. *** without adjusting for sex. Significant associations in bold.

**Table 6:** Sensitivity analysis for occupational history and T2DM and dementia (for the subgroup of 120,299 participants).

Cumulative Occupational Exposure Over Years—Standardised Values (Standardised (P × L) * Years) ***	T2DM OR (95% CI) Univariable	T2DM OR (95% CI) Multivariable *	Dementia OR (95% CI) Univariable	Dementia OR (95% CI) Multivariable **
**Dust**	**1.09 (1.06–1.11)**	1.00 (0.97–1.03)	1.00 (0.54–1.23)	0.95 (0.49–1.21)
**Fumes**	**1.07 (1.04–1.09)**	0.98 (0.95–1.01)	0.99 (0.46–1.19)	0.95 (0.42–1.18)
**Diesel engine exhaust**	**1.07 (1.05–1.09)**	0.98 (0.94–1.01)	1.10 (0.86–1.20)	1.08 (0.83–1.18)
**Mineral dust**	**1.08 (1.05–1.10)**	1.01 (0.97–1.03)	1.07 (0.73–1.23)	1.05 (0.69–1.22)
**Biological dust**	**1.05 (1.02–1.07)**	1.00 (0.97–1.03)	**0.12 (0.00–0.58)**	0.02 (0.00–4.70)

* Adjusted for age, sex, PM_2.5_, ethnicity, Townsend deprivation index, BMI, dietary changes, physical activity, paternal history of diabetes. ** Adjusted for age, sex, PM_2.5_, Townsend deprivation index, dietary changes, physical activity, paternal history of dementia. *** Significant associations in bold.

## Supplementary Materials

The following are available online at https://www.mdpi.com/1660-4601/17/24/9581/s1, FigureS1: Directed acyclic graph showing the model assumptions for T2DM. FigureS2: Directed acyclic graph showing the model assumptions for dementia. Table S1: Percentages used to create exposure score (P × L). Table S2: Observations excluded from the analysis due to incomplete data, in the environmental and main occupational final models for T2DM and dementia. Table S3: Associations between all occupational exposures and T2DM and other potential risk factors by using logistic regression Table S4: Associations between all occupational exposures and dementia and other potential risk factors by using logistic regression. Table S5: Associations between T2DM and dementia and occupational exposure with the interaction term in the model- OR (95%CI) for males and females. Recategorisation of variables and references corresponding to Table S1.

## References

[1] Stone V., Miller M.R., Clift M.J.D., Elder A., Mills N.L., Moller P., Schins R.P.F., Vogel U., Kreyling W.G., Alstrup Jensen K. (2017). Nanomaterials Versus Ambient Ultrafine Particles: An Opportunity to Exchange Toxicology Knowledge. Environ. Health Perspect..

[2] Jovanovic-Andersen Z. (2012). Health effects of long-term exposure to air pollution: An overview of major respiratory and cardiovascular diseases and diabetes. Chem. Ind. Chem. Eng. Q..

[3] WHO (2013). Air Pollution and Cancer.

[4] Donaldson K., Stone V. (2003). Current hypotheses on the mechanisms of toxicity of ultrafine particles. Annali Dell’Istituto Superiore Di Sanita.

[5] Eze I.C., Schaffner E., Fischer E., Schikowski T., Adam M., Imboden M., Tsai M., Carballo D., von Eckardstein A., Kunzli N. (2014). Long-term air pollution exposure and diabetes in a population-based Swiss cohort. Environ. Int..

[6] Teichert T., Vossoughi M., Vierkotter A., Sugiri D., Schikowski T., Schulte T., Roden M., Luckhaus C., Herder C., Kramer U. (2013). Association between traffic-related air pollution, subclinical inflammation and impaired glucose metabolism: Results from the SALIA study. PLoS ONE.

[7] Liu C., Bai Y., Xu X., Sun L., Wang A., Wang T.Y., Maurya S.K., Periasamy M., Morishita M., Harkema J. (2014). Exaggerated effects of particulate matter air pollution in genetic type II diabetes mellitus. Part Fibre Toxicol..

[8] Jung C.R., Lin Y.T., Hwang B.F. (2015). Ozone, particulate matter, and newly diagnosed Alzheimer’s disease: A population-based cohort study in Taiwan. J. Alzheimer’s Dis. JAD.

[9] Moulton P.V., Yang W. (2012). Air pollution, oxidative stress, and Alzheimer’s disease. J. Environ. Public Health.

[10] Calderon-Garciduenas L., Franco-Lira M., Mora-Tiscareno A., Medina-Cortina H., Torres-Jardon R., Kavanaugh M. (2013). Early Alzheimer’s and Parkinson’s disease pathology in urban children: Friend versus Foe responses—It is time to face the evidence. Biomed. Res. Int..

[11] Dimakakou E., Johnston J.H., Streftaris G., Cherrie W.J. (2018). Exposure to Environmental and Occupational Particulate Air Pollution as a Potential Contributor to Neurodegeneration and Diabetes: A Systematic Review of Epidemiological Research. Int. J. Environ. Res. Public Health.

[12] Tsai T.L., Lin Y.T., Hwang B.F., Nakayama S.F., Tsai C.H., Sun X.L., Ma C., Jung C.R. (2019). Fine particulate matter is a potential determinant of Alzheimer’s disease: A systemic review and meta-analysis. Environ. Res..

[13] Seaton A., Tran L., Chen R., Maynard R.L., Whalley L.J. (2020). Pollution, Particles, and Dementia: A Hypothetical Causative Pathway. Int. J. Environ. Res. Public Health.

[14] Allen K.V., Frier B.M., Strachan M.W. (2004). The relationship between type 2 diabetes and cognitive dysfunction: Longitudinal studies and their methodological limitations. Eur. J. Pharmacol..

[15] Van Himbergen T.M., Beiser A.S., Ai M., Seshadri S., Otokozawa S., Au R., Thongtang N., Wolf P.A., Schaefer E.J. (2012). Biomarkers for insulin resistance and inflammation and the risk for all-cause dementia and Alzheimer disease: Results from the Framingham Heart Study. Arch. Neurol..

[16] Biessels G.J., Despa F. (2018). Cognitive decline and dementia in diabetes mellitus: Mechanisms and clinical implications. Nat. Rev. Endocrinol..

[17] Arnold S.E., Arvanitakis Z., Macauley-Rambach S.L., Koenig A.M., Wang H.Y., Ahima R.S., Craft S., Gandy S., Buettner C., Stoeckel L.E. (2018). Brain insulin resistance in type 2 diabetes and Alzheimer disease: Concepts and conundrums. Nat. Rev. Neurol..

[18] Neophytou A.M., Costello S., Picciotto S., Brown D.M., Attfield M.D., Blair A., Lubin J.H., Stewart P.A., Vermeulen R., Silverman D.T. (2019). Diesel Exhaust, Respirable Dust, and Ischemic Heart Disease: An Application of the Parametric g-formula. Epidemiology.

[19] Gallagher L.G., Ray R.M., Li W., Psaty B.M., Gao D.L., Thomas D.B., Checkoway H. (2012). Occupational exposures and mortality from cardiovascular disease among women textile workers in Shanghai, China. Am. J. Ind. Med..

[20] Garshick E., Laden F., Hart J.E., Moy M.L. (2004). Respiratory symptoms and intensity of occupational dust exposure. Int. Arch. Occup. Environ. Health.

[21] De Sio S., Rosati M.V., Cherubini E., Ciarrocca M., Baccolo T.P., Grimaldi F., Caciari T., Tomao E., Tomei F. (2005). Occupational exposure to urban pollutants and plasma insulin. Saudi Med. J..

[22] Tan C., Wang Y., Lin M., Wang Z., He L., Li Z., Li Y., Xu K. (2018). Long-term high air pollution exposure induced metabolic adaptations in traffic policemen. Environ. Toxicol. Pharmacol..

[23] Dimakakou E., Johnston H.J., Streftaris G., Cherrie J.W. (2020). Evaluation of the Suitability of an Existing Job-Exposure Matrix for the Assessment of Exposure of UK Biobank Participants to Dust, Fumes, and Diesel Exhaust Particulates. Int. J. Environ. Res. Public Health.

[24] Sadhra S.S., Kurmi O.P., Chambers H., Lam K.B., Fishwick D., Occupational C.R.G. (2016). Development of an occupational airborne chemical exposure matrix. Occup. Med. (Lond.).

[25] Sudlow C., Gallacher J., Allen N., Beral V., Burton P., Danesh J., Downey P., Elliott P., Green J., Landray M. (2015). UK Biobank: An Open Access Resource for Identifying the Causes of a Wide Range of Complex Diseases of Middle and Old Age. PLoS Med..

[26] Falconer C.L., Cooper A.R., Flint E. (2017). Patterns and correlates of active commuting in adults with type 2 diabetes: Cross-Sectional evidence from UK Biobank. BMJ Open.

[27] Eeftens M., Beelen R., de Hoogh K., Bellander T., Cesaroni G., Cirach M., Declercq C., Dėdelė A., Dons E., de Nazelle A. (2012). Development of Land Use Regression models for PM(2.5), PM(2.5) absorbance, PM(10) and PM(coarse) in 20 European study areas; results of the ESCAPE project. Environ. Sci. Technol..

[28] R Core Team (2019). R: A Language and Environment for Statistical Computing. R Foundation for Statistical Computing, Vienna, Austria. https://www.R-project.org/.

[29] Sadhra S., Kurmi O.P., Sadhra S.S., Lam K.B., Ayres J.G. (2017). Occupational COPD and job exposure matrices: A systematic review and meta-analysis. Int. J. Chronic Obstr. Pulm. Dis..

[30] Akaike H., Lovric M. (2011). Akaike’s Information Criterion. International Encyclopedia of Statistical Science.

[31] Liu C., Yang C., Zhao Y., Ma Z., Bi J., Liu Y., Meng X., Wang Y., Cai J., Kan H. (2016). Associations between long-term exposure to ambient particulate air pollution and type 2 diabetes prevalence, blood glucose and glycosylated hemoglobin levels in China. Environ. Int..

[32] Yang M., Cheng H., Shen C., Liu J., Zhang H., Cao J., Ding R. (2020). Effects of long-term exposure to air pollution on the incidence of type 2 diabetes mellitus: A meta-analysis of cohort studies. Environ. Sci. Pollut. Res. Int..

[33] Fu P., Guo X., Cheung F.M.H., Yung K.K.L. (2019). The association between PM(2.5) exposure and neurological disorders: A systematic review and meta-analysis. Sci. Total Environ..

[34] Lao X.Q., Guo C., Chang L.-Y., Bo Y., Zhang Z., Chuang Y.C., Jiang W.K., Lin C., Tam T., Lau A.K.H. (2019). Long-term exposure to ambient fine particulate matter (PM2.5) and incident type 2 diabetes: A longitudinal cohort study. Diabetologia.

[35] Yang B.-Y., Fan S., Thiering E., Seissler J., Nowak D., Dong G.-H., Heinrich J. (2020). Ambient air pollution and diabetes: A systematic review and meta-analysis. Environ. Res..

[36] Smargiassi A., Sidi E.A.L., Robert L.-E., Plante C., Haddad M., Gamache P., Burnett R., Goudreau S., Liu L., Fournier M. (2020). Exposure to ambient air pollutants and the onset of dementia in Québec, Canada. Environ. Res..

[37] Lee M., Schwartz J., Wang Y., Dominici F., Zanobetti A. (2019). Long-term effect of fine particulate matter on hospitalization with dementia. Environ. Pollut..

[38] Chen H., Kwong J.C., Copes R., Hystad P., van Donkelaar A., Tu K., Brook J.R., Goldberg M.S., Martin R.V., Murray B.J. (2017). Exposure to ambient air pollution and the incidence of dementia: A population-based cohort study. Environ. Int..

[39] Livingston G., Huntley J., Sommerlad A., Ames D., Ballard C., Banerjee S., Brayne C., Burns A., Cohen-Mansfield J., Cooper C. (2020). Dementia prevention, intervention, and care: 2020 report of the Lancet Commission. Lancet.

[40] Tzivian L., Dlugaj M., Winkler A., Weinmayr G., Hennig F., Fuks K.B., Vossoughi M., Schikowski T., Weimar C., Erbel R. (2016). Long-term air pollution and traffic noise exposures and mild cognitive impairment in older adults: A cross-sectional analysis of the Heinz Nixdorf recall study. Environ. Health Perspect..

[41] Cullen B., Newby D., Lee D., Lyall D.M., Nevado-Holgado A.J., Evans J.J., Pell J.P., Lovestone S., Cavanagh J. (2018). Cross-sectional and longitudinal analyses of outdoor air pollution exposure and cognitive function in UK Biobank. Sci. Rep..

[42] Connolly V., Unwin N., Sherriff P., Bilous R., Kelly W. (2000). Diabetes prevalence and socioeconomic status: A population based study showing increased prevalence of type 2 diabetes mellitus in deprived areas. J. Epidemiol. Community Health.

[43] Russ T.C., Stamatakis E., Hamer M., Starr J.M., Kivimäki M., Batty G.D. (2013). Socioeconomic status as a risk factor for dementia death: Individual participant meta-analysis of 86,508 men and women from the UK. Br. J. Psychiatry.

[44] Haffner S.M., D’Agostino R., Saad M.F., Rewers M., Mykkänen L., Selby J., Howard G., Savage P.J., Hamman R.F., Wagenknecht L.E. (1996). Increased insulin resistance and insulin secretion in nondiabetic African-Americans and Hispanics compared with non-Hispanic whites. The Insulin Resistance Atherosclerosis Study. Diabetes.

[45] Rhee E.J. (2015). Diabetes in Asians. Endocrinol. Metab. (Seoul).

[46] Colberg S.R., Sigal R.J., Fernhall B., Regensteiner J.G., Blissmer B.J., Rubin R.R., Chasan-Taber L., Albright A.L., Braun B., American College of Sports M. (2010). Exercise and type 2 diabetes: The American College of Sports Medicine and the American Diabetes Association: Joint position statement. Diabetes Care.

[47] Westreich D., Greenland S. (2013). The Table 2 Fallacy: Presenting and Interpreting Confounder and Modifier Coefficients. Am. J. Epidemiol..

[48] Nordentoft M., Rod N.H., Bonde J.P., Bjorner J.B., Madsen I.E.H., Pedersen L.R.M., Cleal B., Magnusson Hanson L.L., Nexo M.A., Pentti J. (2020). Effort-reward imbalance at work and risk of type 2 diabetes in a national sample of 50,552 workers in Denmark: A prospective study linking survey and register data. J. Psychosom. Res..

[49] Kroenke C.H., Spiegelman D., Manson J., Schernhammer E.S., Colditz G.A., Kawachi I. (2007). Work characteristics and incidence of type 2 diabetes in women. Am. J. Epidemiol..

[50] Hendryx M., Luo J., Chojenta C., Byles J.E. (2019). Exposure to heavy metals from point pollution sources and risk of incident type 2 diabetes among women: A prospective cohort analysis. Int. J. Environ. Health Res..

[51] Huang L.-Y., Hu H.-Y., Wang Z.-T., Ma Y.-H., Dong Q., Tan L., Yu J.-T. (2020). Association of Occupational Factors and Dementia or Cognitive Impairment: A Systematic Review and Meta-Analysis. J. Alzheimer’s Dis..

[52] Helmer C., Letenneur L., Rouch I., Richard-Harston S., Barberger-Gateau P., Fabrigoule C., Orgogozo J.M., Dartigues J.F. (2001). Occupation during life and risk of dementia in French elderly community residents. J. Neurol. Neurosurg. Psychiatry.

[53] Santibáñez M., Bolumar F., García A.M. (2007). Occupational risk factors in Alzheimer’s disease: A review assessing the quality of published epidemiological studies. Occup. Environ. Med..

[54] Park R.M., Schulte P.A., Bowman J.D., Walker J.T., Bondy S.C., Yost M.G., Touchstone J.A., Dosemeci M. (2005). Potential occupational risks for neurodegenerative diseases. Am. J. Ind. Med..

[55] Gunnarsson L.G., Bodin L. (2019). Occupational Exposures and Neurodegenerative Diseases-A Systematic Literature Review and Meta-Analyses. Int. J. Environ. Res. Public Health.

[56] Koeman T., Schouten L.J., van den Brandt P.A., Slottje P., Huss A., Peters S., Kromhout H., Vermeulen R. (2015). Occupational exposures and risk of dementia-related mortality in the prospective Netherlands Cohort Study. Am. J. Ind. Med..

[57] Meo S.A., Al-Khlaiwi T., Abukhalaf A.A., Alomar A.A., Alessa O.M., Almutairi F.J., Alasbali M.M. (2020). The Nexus between Workplace Exposure for Wood, Welding, Motor Mechanic, and Oil Refinery Workers and the Prevalence of Prediabetes and Type 2 Diabetes Mellitus. Int. J. Environ. Res. Public Health.

[58] Ritz B., Hoffmann B., Peters A. (2019). The Effects of Fine Dust, Ozone, and Nitrogen Dioxide on Health. Dtsch. Arzteblatt Int..

[59] Peters S., Vermeulen R., Portengen L., Olsson A., Kendzia B., Vincent R., Savary B., Lavoué J., Cavallo D., Cattaneo A. (2016). SYN-JEM: A Quantitative Job-Exposure Matrix for Five Lung Carcinogens. Ann. Occup. Hyg..

[60] Schraufnagel D.E. (2020). The health effects of ultrafine particles. Exp. Mol. Med..

