# Supplementary material for: Is Environmental and Occupational Particulate Air Pollution Exposure Related to Type-2 Diabetes and Dementia? A Cross-Sectional Analysis of the UK Biobank
Source: Int J Environ Res Public Health. 2020 Dec 21;17(24):9581. doi: 10.3390/ijerph17249581 (PMC7767456; doi:10.3390/ijerph17249581)
Supplement: Supplementary file 1 [file ijerph-17-09581-s001.pdf]

Supplementary Material

Is environmental and occupational particulate air pollution exposure related to type-2 diabetes and dementia? A cross-sectional analysis of the UK Biobank

Eirini Dimakakou [1], Helinor J. Johnston[1], George Streftaris [2] and John W. Cherrie [1,3]

[1] Institute of Biological Chemistry, Biophysics and Bioengineering, Heriot-Watt University, Edinburgh EH14 4AS, UK

[2] Maxwell Institute for Mathematical Sciences, School of Mathematical and Computer Sciences, Heriot-Watt University, Edinburgh EH14 4AS, UK

[3] Institute of Occupational Medicine (IOM), Riccarton, Edinburgh EH14 4AP, UK

Table of Contents

1. Strobe guidelines followed Pg. 1
2. Figures (DAGs) Pg. 2
3. Tables Pg. 4
4. Recategorisation of covariates Pg. 7
5. References Pg. 8

1. **Reporting follows STrengthening the Reporting of OBservational studies in Epidemiology (STROBE) guidelines.**

.

2. Figures

Figure S1: Directed acyclic graph showing the model assumptions for T2DM.

The red arrow represents assumed causal relationship between particulate air pollution exposure and T2DM. Blue arrows represent implied confounding paths before any covariate adjustment has been applied. Adjustments were made for all the variables, except for BP that was a potential mediator.

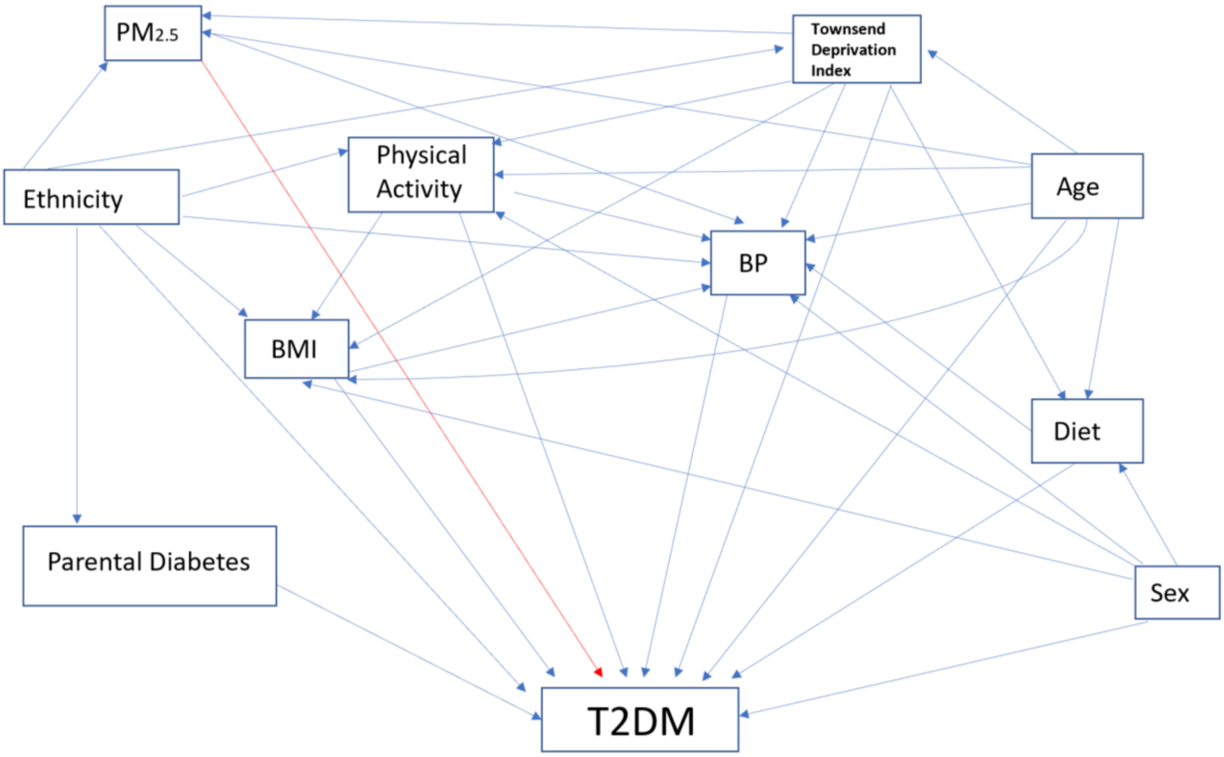

Figure S2: Directed acyclic graph showing the model assumptions for dementia.

The red arrow represents assumed causal relationship between particulate air pollution exposure and dementia. Blue arrows represent implied confounding paths before any covariate adjustment has been applied. Adjustments were made for all the variables, except for BP that was a potential mediator.

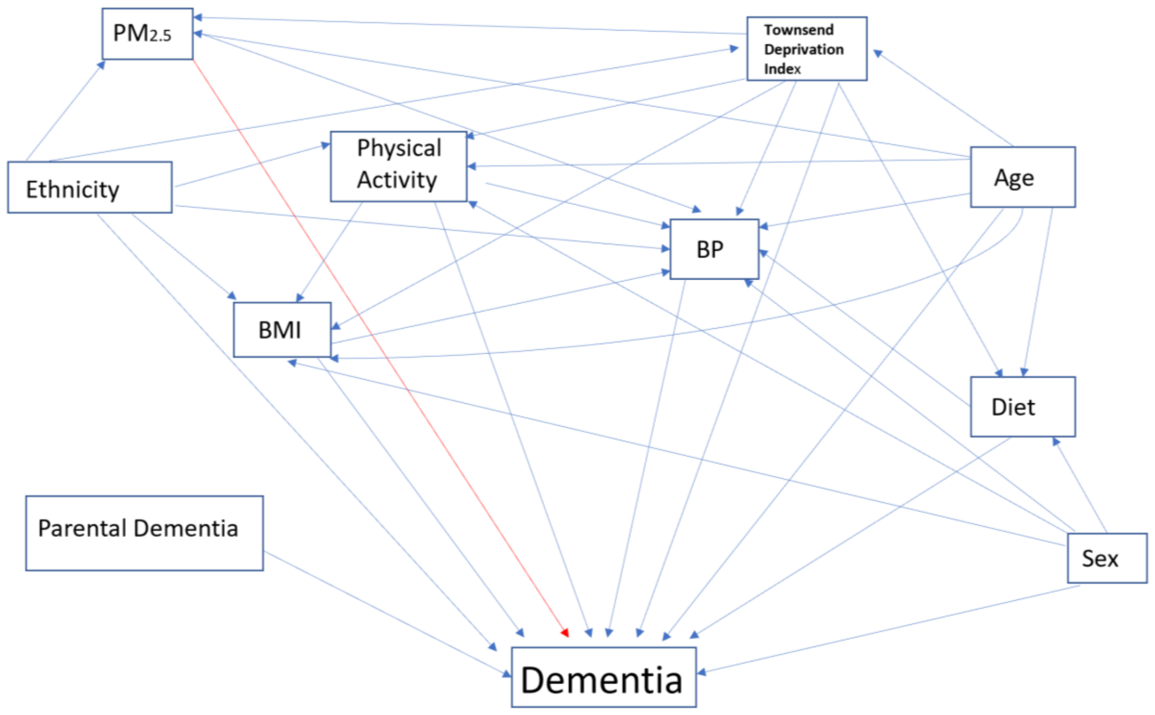

3. Tables

Table S1. Percentages used to create exposure score (P×L) [1-3]

	**Proportion (P)**	**Level (L)**
**High**	75% (0.75)	150% (1.50)
**Medium**	35% (0.35)	30% (0.30)
**Low**	12.5% (0.125)	6% (0.06)
**Unexposed**	2.5% (0.025)	0% (0.00)

TableS2. Observations excluded from the analysis due to incomplete data, in the environmental and main occupational final models for T2DM and dementia

**Environmental model**	T2DM	67,534
	Dementia	65,403
**Occupational model**	T2DM	227,499 *
	Dementia	226,617 *

* Occupational information was missing

Table S3. Associations between all occupational exposures and T2DM and other potential risk factors by using logistic regression

**T2DM**	**O.R. (95% CI)** **Dust**	**O.R. (95% CI)** **Fumes**	**O.R. (95% CI)** **Diesel**	**O.R. (95% CI)** **Mineral Dust**	**O.R. (95% CI)** **Biological Dust**
**(P×L)** (1)					
Level.2	0.93 (0.88-0.98)	1.01 (0.95-1.07)	1.07 (0.99-1.15)	0.91 (0.86-0.97)	0.95 (0.89-1.02)
**PM$_{2.5}$**	1.01 (0.99-1.04)	1.01 (0.99-1.04)	1.01 (0.99-1.04)	1.01 (0.99-1.04)	1.01 (0.99-1.04)
**Sex** (female)					
Male	1.81 (1.73-1.90)	1.80 (1.71-1.89)	1.78 (1.70-1.87)	1.83 (1.74-1.92)	1.79 (1.71-1.88)
**Age**	1.08 (1.08-1.08)	1.08 (1.08-1.08)	1.08 (1.08-1.08)	1.08 (1.08-1.08)	1.08 (1.08-1.08)
**Ethnic background** (White)					
Asian	3.97 (3.60-4.37)	3.96 (3.60-4.36)	3.96 (3.60-4.36)	3.95 (3.59-4.35)	3.97 (3.61-4.37)
Black	1.70 (1.49-1.93)	1.69 (1.48-1.92)	1.69 (1.48-1.92)	1.68 (1.48-1.91)	1.70 (1.49-1.93)
Mixed	1.17 (0.87-1.55)	1.17 (0.87-1.55)	1.17 (0.87-1.55)	1.17 (0.87-1.55)	1.17 (0.87-1.55)
Other	1.63 (1.37-1.92)	1.62 (1.37-1.92)	1.63 (1.37-1.92)	1.62 (1.36-1.91)	1.63 (1.37-1.92)
**Townsend deprivation** (1)					
2	1.07 (0.99-1.16)	1.07 (0.99-1.16)	1.07 (0.99-1.16)	1.07 (0.99-1.16)	1.07 (0.99-1.16)
3	1.13 (1.05-1.22)	1.13 (1.05-1.22)	1.13 (1.05-1.22)	1.13 (1.05-1.22)	1.13 (1.05-1.22)
4	1.18 (1.09-1.27)	1.18 (1.09-1.27)	1.17 (1.09-1.27)	1.18 (1.09-1.27)	1.18 (1.09-1.27)
5 (most deprived)	1.32 (1.22-1.43)	1.31 (1.21-1.42)	1.31 (1.21-1.42)	1.31 (1.21-1.42)	1.31 (1.21-1.42)
**BMI** (<18.5)					
>=30	17.67 (7.49-57.40)	17.63 (7.47-57.27)	17.57 (7.45-57.08)	17.71 (7.51-57.56)	17.63 (7.47-57.26)
25-29.9	6.47 (2.74-21.04)	6.46 (2.74-21.00)	6.45 (2.73-20.95)	6.49 (2.75-21.09)	6.46 (2.74-21.00)
18.5-24.9	3.09 (1.31-10.04)	3.08 (1.30-10.03)	3.08 (1.30-10.02)	3.09 (1.31-10.06)	3.08 (1.30-10.03)
**Dietary changes** (No)					
Unknown	1.80 (1.08-2.82)	1.78 (1.07-2.79)	1.77 (1.06-2.78)	1.80 (1.08-2.82)	1.78 (1.07 -2.80)
Yes, because of illness	13.57 (12.87-14.32)	13.57 (12.86-14.31)	13.57 (12.86-14.31)	13.57 (12.86-14.31)	13.57 (12.87-14.32)
Yes, because of other	1.46 (1.37-1.56)	1.46 (1.38-1.56)	1.46 (1.38-1.56)	1.46 (1.37-1.56)	1.46 (1.38-1.56)
**Physical activity** (Low)					
High	0.75 (0.70-0.79)	0.74 (0.70-0.79)	0.74 (0.70-0.79)	0.75 (0.70-0.79)	0.74 (0.70-0.79)
Moderate	0.85 (0.80-0.89)	0.85 (0.81-0.89)	0.85 (0.81-0.89)	0.85 (0.81-0.89)	0.85 (0.81-0.89)
**Father's diabetes history** (No)					
Do not know	1.28 (1.16-1.40)	1.27 (1.16-1.40)	1.27 (1.15-1.40)	1.27 (1.16-1.40)	1.27 (1.16-1.40)
Prefer not to answer	0.76 (0.33-1.57)	0.76 (0.32-1.56)	0.76 (0.32-1.56)	0.76 (0.32-1.57)	0.76 (0.32-1.56)
Yes	2.42 (2.28-2.57)	2.42 (2.28-2.57)	2.42 (2.28-2.57)	2.42 (2.28-2.57)	2.42 (2.28-2.57)
**Mother's diabetes history** (No)					
Do not know	0.99 (0.87-1.13)	0.99 (0.87-1.12)	0.99 (0.87-1.12)	0.99 (0.87-1.13)	0.99 (0.87-1.13)
Prefer not to answer	0.88 (0.33-2.02)	0.87 (0.33-2.00)	0.87 (0.33-2.01)	0.87 (0.32-2.00)	0.88 (0.33-2.01)
Yes	1.07 (1.02-1.12)	1.07 (1.02-1.12)	1.07 (1.02-1.12)	1.07 (1.02-1.12)	1.07 (1.02-1.12)

Table S4. Associations between all occupational exposures and dementia and other potential risk factors by using logistic regression

**Dementia**	**O.R. (95% CI)**	**O.R. (95% CI)**	**O.R. (95% CI)**	**O.R. (95% CI)**	**O.R. (95% CI)**
	**Dust**	**Fumes**	**Diesel**	**Mineral Dust**	**Biological Dust**
**(P×L)** (1)					
Level.2	1.02 (0.71-1.45)	1.25 (0.84-1.84)	0.98 (0.56-1.62)	1.02 (0.66-1.51)	0.97 (0.58-1.52)
**$PM_{2.5}$**	1.14 (0.97-1.33)	1.15 (0.98-1.33)	1.14 (0.97-1.33)	1.14 (0.97-1.33)	1.14 (0.97-1.33)
**Sex** (female)					
Male	2.00 (1.42-2.84)	1.90 (1.33-2.72)	2.01 (1.42-2.86)	1.99 (1.41-2.85)	2.00 (1.43-2.83)
**Age**	1.10 (1.07-1.13)	1.10 (1.07-1.13)	1.10 (1.07-1.13)	1.10 (1.07-1.13)	1.10 (1.07-1.13)
**Townsend deprivation** (1)					
2	0.69 (0.35-1.30)	0.68 (0.35-1.30)	0.69 (0.35-1.30)	0.69 (0.35-1.30)	0.69 (0.35-1.30)
3	1.26 (0.73-2.21)	1.25 (0.72-2.20)	1.26 (0.73-2.21)	1.26 (0.73-2.21)	1.26 (0.73-2.21)
4	1.68 (1.00-2.91)	1.68 (1.00-2.89)	1.69 (1.00-2.91)	1.69 (1.00-2.91)	1.69 (1.00-2.91)
5 (most deprived)	1.59 (0.91-2.84)	1.58 (0.90-2.82)	1.59 (0.91-2.84)	1.59 (0.91-2.84)	1.59 (0.91-2.84)
**Dietary changes** (No)					
Unknown	2.77 (0.14-14.59)	2.79 (0.15-14.56)	2.78 (0.14-14.62)	2.77 (0.14-14.60)	2.79 (0.15-14.66)
Yes, because of illness	1.94 (1.21-3.00)	1.93 (1.21-2.99)	1.94 (1.21-3.00)	1.94 (1.21-3.00)	1.94 (1.21-3.00)
Yes, because of other	1.44 (0.99-2.07)	1.44 (0.99-2.07)	1.44 (0.99-2.07)	1.44 (0.99-2.07)	1.44 (0.99-2.07)
**Physical activity** (Low)					
High	0.85 (0.56-1.27)	0.85 (0.56-1.27)	0.85 (0.56-1.27)	0.85 (0.56-1.27)	0.85 (0.56-1.28)
Moderate	0.69 (0.47-1.01)	0.70 (0.48-1.02)	0.69 (0.47-1.01)	0.69 (0.47-1.01)	0.69 (0.47-1.01)
**Father's dementia history** (No)					
Do not know	1.79 (0.99-3.00)	1.77 (0.98-2.98)	1.79 (0.99-3.01)	1.79 (0.99-3.01)	1.79 (0.99-3.01)
Prefer not to answer	0.93 (0.04-7.65)	0.92 (0.04-7.53)	0.93 (0.04-7.67)	0.93 (0.04-7.64)	0.93 (0.04-7.64)
Yes	1.85 (0.99-3.16)	1.86 (1.00-3.17)	1.85 (0.99-3.16)	1.85 (0.99-3.16)	1.85 (0.99-3.16)
**Mother's dementia history** (No)					
Do not know	0.82 (0.28-1.85)	0.81 (0.28-1.83)	0.82 (0.28-1.85)	0.82 (0.28-1.85)	0.82 (0.28-1.85)
Prefer not to answer	25.85 (4.92-82.74)	25.58 (4.89-81.66)	25.94 (4.94-82.85)	25.93 (4.94-82.84)	26.04 (4.96-83.23)
Yes	0.95 (0.52-1.60)	0.95 (0.52-1.59)	0.95 (0.52-1.60)	0.95 (0.52-1.60)	0.95 (0.52-1.60)

Table S5. Associations between T2DM and dementia and occupational exposure with the interaction term in the model- OR (95%CI) for males and females

		T2DM		Dementia	
		**O.R. (95% CI)**	**p-values**	**O.R. (95% CI)**	**p-values**
Dust	F	1.10 (1.01-1.21)		1.37 (0.71-2.49)	
	M	0.77 (0.69-0.86)	0.020762 *	0.65 (0.31-1.41)	0.269774
Fumes	F	1.04 (0.90-1.21)		0.97 (0.23-2.64)	
	M	0.96 (0.82-1.13)	0.626165	1.35 (0.45-5.83)	0.63588
Diesel	F	0.98 (0.76-1.24)		1.79 (0.29-5.78)	
	M	1.10 (0.85-1.42)	0.487586	0.51 (0.13-3.34)	0.38677
Mineral dust	F	0.99 (0.86-1.15)		1.39 (0.48-3.21)	
	M	0.90 (0.77-1.06)	0.195160	0.68 (0.26-2.14)	0.471116
Biological dust	F	1.13 (1.02-1.24)		1.25 (0.59-2.41)	
	M	0.73 (0.64-0.83)	2.69e-06 ***	0.63 (0.24-1.66)	0.34333

F: Females
M: Males

4. Recategorisation of covariates

Age was constructed from variable 34 from the UK Biobank.

BMI was constructed from variable 21001, which had BMI information from 4 instances (initial assessment visit, first repeat assessment, imaging visit and first repeat imaging visit). A mean was obtained from all the measurements and then this variable was recategorised in 4 levels (level 1: BMI < 18.5 (underweight), level 2: BMI 18.5-24.9 (healthy), level 3: BMI 25-29.9 (overweight) and level 4: BMI >= 30 (obese)).

Townsend deprivation index was based on variable 189 and on the preceding national census output areas. Each participant was assigned a score corresponding to the output area in which their postcode was located, and 5 levels were constructed, with level 1 corresponding to the least deprived areas and level 5 to the most deprived areas.

Cholesterol levels is a risk factor for dementia, and blood pressure medication is a risk factor for both diabetes and dementia, therefore the variables 6177 (information only for males) and 6153 (information only for females) from UK Biobank were used to extract this information. Firstly, all the columns of each variable were merged to one, as 12 different measurements were available, and then recategorised, to obtain one new variable that represented cholesterol medication (Yes/No/Unknown) and another one that represented blood pressure medication (Yes/No/Unknown).

To investigate diet as a risk factor, we recategorised variable 1538, which represents major dietary changes in the last 5 years. We merged the information from all the instances, by keeping firstly the answer “Yes, because of illness” and then the answer “Yes, because of

other reasons" and then the "No" and lastly the "Prefer not to answer" responds. Therefore, we created a variable with 4 levels.

Stroke, which is a risk factor for dementia, was extracted from data fields 42006. This information was algorithmically defined from Hospital Episode data, so it was gathered at specific instances, but it is continually updated from the NHS linkage systems and offers the participant's information from 1975 up to date. Thus, the dates in this variable were replaced with a "Yes" and all the other with a "No".

Physical activity was extracted from variable 22040, which is the summed Total Metabolic Equivalent Task (MET) minutes per week for all activity, including walking, moderate and vigorous activity. We created 3 different levels, low, moderate, and high according to IPAQ (International Physical Activity Questionnaire) guidelines.

From variables 20107 and 20110 we extracted information about parental illnesses (illnesses of father and mother accordingly). From 31 columns, that provided a follow-up information for parental diabetes and dementia history, we created 2 columns for parental diabetes history (father and mother) and 2 columns for parental history of dementia. "Yes" corresponds to diabetes or dementia family history, "No" corresponds to other conditions and then "Prefer not to answer" and "Do not know".

5. References

1. Kauppinen, T.; Heikkilä, P.; Plato, N.; Woldbaek, T.; Lenvik, K.; Hansen, J.; Kristjansson, V.; Pukkala, E. Construction of job-exposure matrices for the Nordic Occupational Cancer Study (NOCCA). *Acta oncologica (Stockholm, Sweden)* **2009**, *48*, 791-800.
2. Offermans, N.S.; Vermeulen, R.; Burdorf, A.; Peters, S.; Goldbohm, R.A.; Koeman, T.; van Tongeren, M.; Kauppinen, T.; Kant, I.; Kromhout, H., et al. Comparison of expert and job-exposure matrix-based retrospective exposure assessment of occupational carcinogens in The Netherlands Cohort Study. *Occup Environ Med* **2012**, *69*, 745-751.
3. Sadhra, S.S.; Kurmi, O.P.; Chambers, H.; Lam, K.B.; Fishwick, D.; Occupational, C.R.G. Development of an occupational airborne chemical exposure matrix. *Occup Med (Lond)* **2016**, *66*, 358-364.
